# Magilock: a reliable control triggering method in multi-channel eye-control systems

**DOI:** 10.3389/fnhum.2024.1365838

**Published:** 2024-03-22

**Authors:** Niu Ya-Feng, He Jia-Xin, Liu Jin

**Affiliations:** Department of Industrial Design, School of Mechanical Engineering, Southeast University, Nanjing, China

**Keywords:** eye control, multi-channel interaction, Midas touch, lock time, unlock time

## Abstract

Eye-tracking technology brings a different human-computer interaction experience to users because of its intuitive, natural, and hands-free operation characteristics. Avoiding the Midas touch problem and improving the accuracy of interaction are among the main goals of the research and development of eye-control systems. This study reviews the methods and limitations of research on avoiding the Midas touch problem. For typical control clicking operations with low fault tolerance, such as mode switching and state selection in an eye-control system, this study proposes Magilock, a more reliable control triggering method with a high success rate in multi-channel eye-control systems. Magilock adds a control pre-locked mechanism between the two interactive steps of eye-control channel positioning control and other interactive channel triggering controls in the multi-channel eye-control system. This effectively avoids incorrect control triggering caused by multi-channel coordination disorder and gaze-point drift. This study also conducted ergonomic experiments to explore the lock and unlock times of the control pre-locked mechanism in Magilock. Taking into account the experimental data and subjective evaluation of the participants, we recommend setting the lock time and the unlock time of Magilock to 200 ms.

## 1 Introduction

Increasingly complex human-computer systems have put forward higher requirements for operators, making people realize the value of human-computer interaction research. The development and application of interactive methods, such as voice control, gesture recognition, brain-computer interfaces, eye tracking, and emotion recognition, have improved users’ human-computer interaction experience in different scenarios (Cowie et al., 2001; Niu et al., 2023). The eye-control system is a human-computer interaction system in which the user outputs control instructions to the computer through eye movements, such as gaze, eye gestures, smooth tracking, and eye blinks. In recent years, the cost of eye-tracking equipment has decreased, and the eye-control system has been widely used in many fields. For example, the eye-control system frees the user’s hands and is particularly suitable for patients with hand dysfunction and situational dysfunction whose hands are occupied by other interactive tasks (Göbel et al., 2013). Eye-control systems are also suitable for gaming and educational research.

In the eye-control system, the Midas touch problem is one of the main problems affecting the accuracy of user interaction. ‘Midas touch’ means that when the user’s gaze point falls on the interactive area of the screen, the system is unable to accurately determine whether the user is browsing for information or interacting with the system, thus incorrectly activating interaction commands that are not expected by the user (Jacob, 1995). In previous studies, methods for avoiding the Midas touch problem can be divided into three categories. The first category is optimizing the design of the interface of the eye-control system, such as designing a separate expansion form for the interface menu or bringing in a fisheye lens to highlight the interactive elements of the interface. The second category is optimizing eye interaction actions, such as research on combining and matching various types of eye movements or ergonomics research on the execution time or effective interface range of different eye movements. These two types of research have been conducted on single-channel eye-control systems, which have improved the success rate of eye movement interaction and reduced the Midas touch problem. However, because the eye channel is responsible for both information browsing and interaction command output, the single-channel eye-control system may still misinterpret the user’s intention. Therefore, inspired by MagicPoint (Zhai et al., 1999), as the third category of methods for solving the Midas touch problem, researchers have attempted to add other interaction channels to the eye-control system, which can be used to independently perform the task of outputting user interaction commands. These studies on eye control interaction mainly focus on the technical implementation of the system, the design of control interfaces, and the characteristics of eye movement (Wang et al., 2024), mainly involving computer vision-based methods (Choi et al., 2011; Attivissimo et al., 2023; Hong et al., 2023). In multi-channel eye-control systems, the eye interaction channel only needs to locate the user’s gaze point and no longer outputs interaction commands, which is more effective in avoiding the Midas touch problem. However, the new channels cause new human-computer interaction problems. For example, during the frequent use of the system, the two channels may be misordered, which reduces the accuracy of the user’s operation.

Based on current research, eye-control systems already have a relatively high control trigger success rate, which can satisfy users’ needs in most scenarios. However, when outputting some key operation commands, such as menu selection or mode selection, a small probability of control mis-triggering may still occur. The cost of correcting such mistakes is high and may affect user experience. Therefore, this study proposed a more reliable control selection method for multi-channel eye-control systems, called Magilock. Based on previous research results, we chose ‘dwell time’ as the control locking method, which ensured natural and smooth interactions of the entire process.

This study conducted ergonomic experiments on the lock and unlock times of the pre-locked mechanism in Magilock. Based on the experimental results and subjective evaluation of the participants, the recommended lock and unlock times were determined. The proposed Magilock can effectively improve the correct triggering rate of key commands or controls in the eye-control system, ensuring the user’s interactive experience. Magilock provides a reference for the design of the trigger forms of key commands in multi-channel eye-control systems.

In addition, this work integrates tactile channels to undertake some interactive tasks, which can alleviate or solve the Midas touch caused by the overlapping behavioral characteristics of human eye “browsing” and “control” in eye-control systems. The addition of tactile channels will reduce the attention time and visual stimulation on the interface, and improve the comfort of the eyes. This research work has important academic value for enriching and developing the theory of human-computer interaction in eye-control systems, interface design specifications, and ergonomic evaluation systems. It will also provide new ideas and research fields for the multi-channel human-computer interaction technology and advanced interaction technology.

## 2 Related works

Previous research can be classified into research based on single-channel eye-control systems and research based on multi-channel eye-control systems. Studies on single-channel eye-control systems have mostly improved the performance of eye-control systems by optimizing the system interface or designing eye interaction actions. Multi-channel eye-control systems can add other interaction channels to improve the system performance. In this section, we review related studies and discuss their limitations.

### 2.1 Eye-control system interface optimization

In research on menu design for eye-control systems, to avoid the Midas touch problem, Tien and Atkins (2008) set up a separate selection button for the menu of the eye-control system. After gazing at the target option of the menu, the user needs to perform an additional scanning action on the button to trigger the menu option. The additional sweeping action tires the user, and after a certain time of use, the correctness of the user’s menu selection may become low. Kammerer et al. (2008) developed a semi-circular menu expansion format for eye-control systems and found that the selection time and correctness of the semi-circular menu were significantly better than those of the traditional linear menu expansion format. However, this semi-circular menu expansion may not be suitable for scenarios with complex menus (Kammerer et al., 2008). Elmadjian and Morimoto (2021) developed a gaze-path-based ‘GazeBar’ menu. Although it may not avoid the ‘Midas touch’ problem, it is less costly for the user to correct errors. In research on the interface and control layout of the eye-control system, for different eye movement interaction modes, such as gaze, blinking, eye gesture, and smooth tracking, Niu et al. (2019), Ya-feng et al. (2022), and Niu et al. (2023) conducted ergonomic investigations for the size and interval of the system interaction controls, as well as the display and color effects of the interface information, which promoted the construction of the interface design parameter framework of the eye-control system. Yi-yan et al. (2023) further investigated the effects of a smooth tracking path and target size on the user interaction performance in eye-control systems and provided corresponding design suggestions. Zuo et al. (2023) investigated the user preference of the interface and graphical display under the eye-control system with a monocular eye gesture mode of interaction. Applying interface enhancement display techniques, such as fisheye lenses, to eye-control systems can also improve the correctness of the user’s eye clicks, although fisheye lenses may affect the user’s information browsing (Ashmore et al., 2005).

### 2.2 Eye interaction movement research

In research on eye interaction actions and forms, Istance et al. (2008) proposed the ‘Snap Clutch’, which allows the user to switch modes in the eye-control system by rapidly scanning the screen in different directions and switch to non-interactive modes to avoid the Midas touch problem when they do not need to interact with the screen. However, involuntary eye gestures may also lead to incorrect mode switching in eye-control systems. Ramirez Gomez et al. (2021) developed an interaction method called ‘Gaze + Hold,’ which can be used for dragging and box-selecting controls in eye-controlled interfaces. This method requires the user to close one eye before interacting with the computer, which may avoid the Midas touch problem; however, it is not natural and not suitable for other interface control commands. Ma et al. (2023) proposed a control trigger method in eye-control systems that combines gaze and blink. In their research, the users need to perform a voluntary blink to trigger the control after selecting the target control by gazing, which improves the correctness of the control selection rate of the eye-control system; however, an involuntary eye blink may still cause the Midas touch problem. Yi-yan et al. (2023) further optimized the temporal and spatial properties of the ‘Gaze + Blink’ system to reduce the impact of the Midas touch problem.

### 2.3 Eye-control systems with other interaction channels

Zhai et al. (1999) proposed MAGIC POINT to introduce a manual into the eye-control system, which improved the accuracy and efficiency of the system. Inspired by this, researchers attempted to combine other interaction channels with eye-control systems to improve system performance (Surakka et al., 2004). In their study on the eye-control system combined with head channels, Špakov et al. (2014) introduced micro-movements of the head into the eye-control system, which allowed the user to change the target pointed at by the eye through head movements, thereby improving the system pointing accuracy for small targets. The HMAGIC system proposed by Kurauchi et al. (2015) also combined head movements with the eye-control system, which improved the accuracy of the system’s judgment of the user’s gaze intention. The BimodalGaze developed by Sidenmark et al. (2020) realized the smooth switching between eye-controlled pointing and head adjustments of the system, improving the usability of the head-eye pointing system. Miniotas et al. (2006) combined voice control with the eye-control system to improve the accuracy of pointing at small target controls. The EyeTAP system proposed by Parisay et al. (2020) replaced voice control with sound pulse recognition to render the system more robust under environmental noise disturbances. In addition, some researchers introduced special facial interactions, such as breathing and lip-speaking, into the eye-control system (Su et al., 2021; Onishi et al., 2022).

In research on the eye-control systems combined with hand channels, Chatterjee et al. (2015) introduced hand gesture recognition technology into the eye-control system in which different gestures of the human hand can be used for different system commands. Pfeuffer et al. (2017) proposed the ‘Gaze + Pinch’ method that can be used in virtual reality, and through the combination of the eye and the hand, users can complete a variety of tasks, such as 3D object control, map navigation, and image zooming. Similarly, in the field of virtual reality, Schweigert et al. proposed a control selection method called ‘Eyepointing.’ Eye pointing combines eye tracking with finger pointing to improve the accuracy of control selection tasks (Schweigert et al., 2019). In research on eye-control systems combined with foot interaction channels, Çöltekin et al. (2016) developed a Geographic Information System (GIS) controlled by eye gaze and feet movement, and Hatscher et al. (2017) developed the GazeTap system that allowed physicians to interact with medical images in minimally invasive interventions through eye and foot movements.

### 2.4 Eye-control systems with other interaction channels

In summary, in research on single-channel eye-control systems, researchers often improve the interaction efficiency of the system through interface design and optimization of eye interaction movements. However, because the eye channel in the system is used as an information receiving channel to undertake the task of interface information viewing and as a command output channel to undertake the task of interactive command output, these characteristics make the single-channel eye-control system not completely and correctly determine the user’s eye-gaze intention, resulting in the Midas touch problem. Moreover, researchers attempted to combine different interaction channels in multi-channel eye-control systems. This type of research is primarily focused on the development and realization of the system, and the use of the system is limited. Moreover, the introduction of new interaction channels caused new interaction problems. Recently, few scholars have conducted generalized interaction research and ergonomics research that can be applied to various types of multi-channel eye-control systems, and these types of research can improve the interaction efficiency of the system and user experience. This study aimed to make some contributions in this aspect.

In an eye-control system, mode selection, state switching, and other control commands, which we call key commands, require high triggering accuracy. When key commands are mis-triggered, users need to pay a high error-correction cost, which may significantly affect their interaction experience. Therefore, based on research on multi-channel eye-control systems, this study proposed Magilock, an interaction mode for triggering key commands in eye-control systems. Magilock requires the user to perform an additional confirmation step before the trigger operation to improve the correct triggering rate of key commands in multi-channel eye-control systems. As an interaction mechanism that can be generalized to eye-control systems with different interaction channels, Magilock provides a reference for designing key command-triggering formats for multi-channel eye-control systems.

## 3 System design

### 3.1 Software and hardware systems

**Figure 1:**
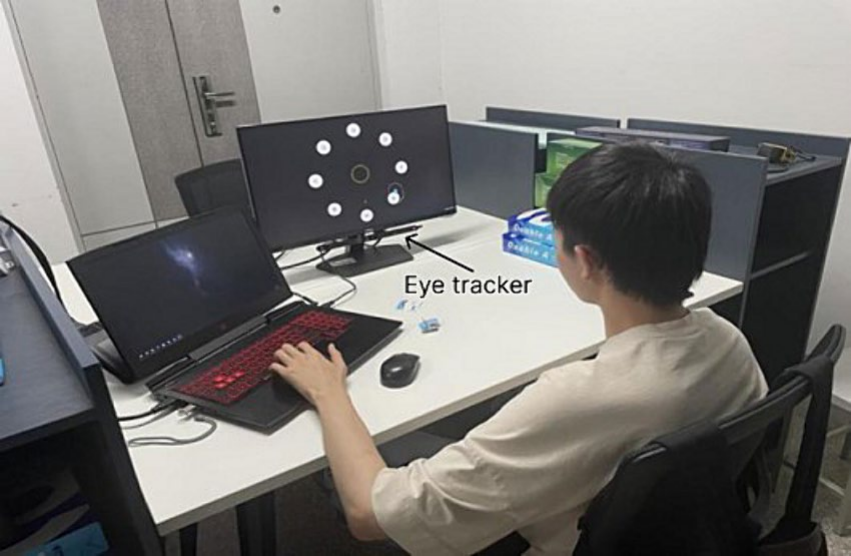
The experimental scenario.

The hardware of the Magilock eye-control system developed in this study consisted of a computer and an eye tracker. The eye tracker used in this study was Tobii Eye Tracker 5, which was used to show the coordinates of the user’s eye gaze points on the computer screen. The computer was used to run the experimental program and receive and process user interaction commands. The display size was 15.6 inches with a resolution of 1920*1080px. Based on the Tobii Unity SDK for Desktop, we developed an experimental program in Unity 3D using C# language. The experimental scenario is shown in Figure 1.

### 3.2 Interactive processes

To avoid incorrect triggering of controls in a multi-channel eye-control system due to mismatch or disorder of different channels and improve the accuracy of triggering key commands in the eye-control system, we proposed the Magilock interaction strategy in this study. This interaction strategy adds a pre-locked mechanism to the interaction flow of the multi-channel eye-control system between the eye channel positioning control process and the other channel triggering control processes. In this study, the control is locked when the user gazes at it for a certain period of time. This time is defined as the lock time in Magilock. If the user’s gaze point moves away from the control before it is locked, the pre-locked mechanism does not take effect. After the control is locked by the user, if the user wants to unlock it, they need to look away from the locked control for a certain period of time. This time is defined as the unlock time of the control. After the pre-locked mechanism is introduced into the multi-channel eye-control system, the user’s operation commands can only be directed to the locked controls. When there is no locked control, user commands are invalid. This pre-locked mechanism avoids incorrect trigger of the control caused by gaze-point drift or other factors (Rajanna and Hammond, 2016).

In the Magilock mechanism, the controls in the eye-control system have three states: normal state (unlocked state), locking state, and locked state. To allow the user to clearly recognize the state of the control, we designed a distinct form of visual feedback for the interactive controls in the experimental program. Controls are white in the normal state (unlocked state). When the user looks at a control, it starts locking and turns blue. When the control is locked, it turns green.

**Figure 2:**
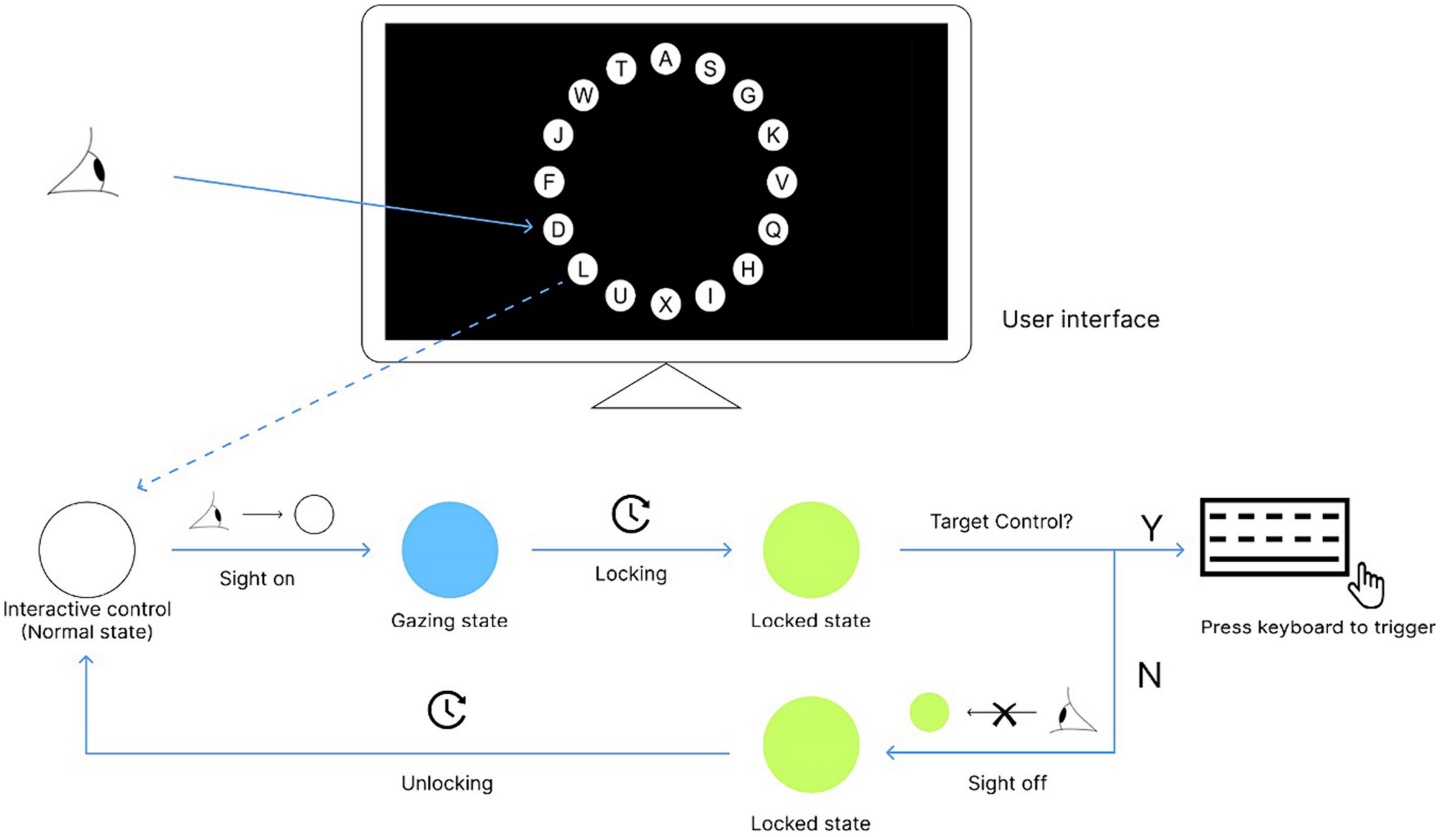
Schema for Magilock.

The trigger flow of the control in the Magilock mechanism is shown in Figure 2. The trigger process of the eye-control system in the Magilock mechanism can be described as follows: (1) The user browses the interface and moves the gaze point to the target control that needs to be triggered, and the control enters the locking state (changing from white to blue). (2) The user looks at the interactive control for a period of time to lock the control (from blue to green). (3) The user triggers the control through other interaction channels to complete the interaction process.

In this study, we chose to combine the hand interaction channel with the eye-control system, and the control was triggered by pressing the space bar on the computer keyboard with the hand. This trigger form is common, representative, and inexpensive. This study focuses on the Magilock pre-locked mechanism introduced in a multi-channel eye-control system, rather than the type of interaction channel to be combined with the eye-control system. Therefore, in this study, a representative and low-cost keyboard press was selected as the control trigger form. In subsequent research or applications, different interaction channels and trigger forms can be replaced according to the users’ needs and application scenarios.

## 4 Magilock lock time research

### 4.1 Purpose

This experiment investigates the value of the lock time in the Magilock pre-locked mechanism. Because the Magilock mechanism is applied to a multi-channel eye-control system, the control lock time under this mechanism is different from the dwell time that causes the control to be triggered in a single-channel eye-control system (Majaranta et al., 2006). The previous studies on the dwell time for single-channel eye-control systems may not be suitable for setting the lock time for the Magilock mechanism. Therefore, in this section, an ergonomic experiment was conducted to investigate the optimal lock time for the Magilock mechanism. It is expected that the optimal lock time for the Magilock mechanism will be determined to ensure the efficiency and experience of user interaction.

### 4.2 Design

**Figure 3:**
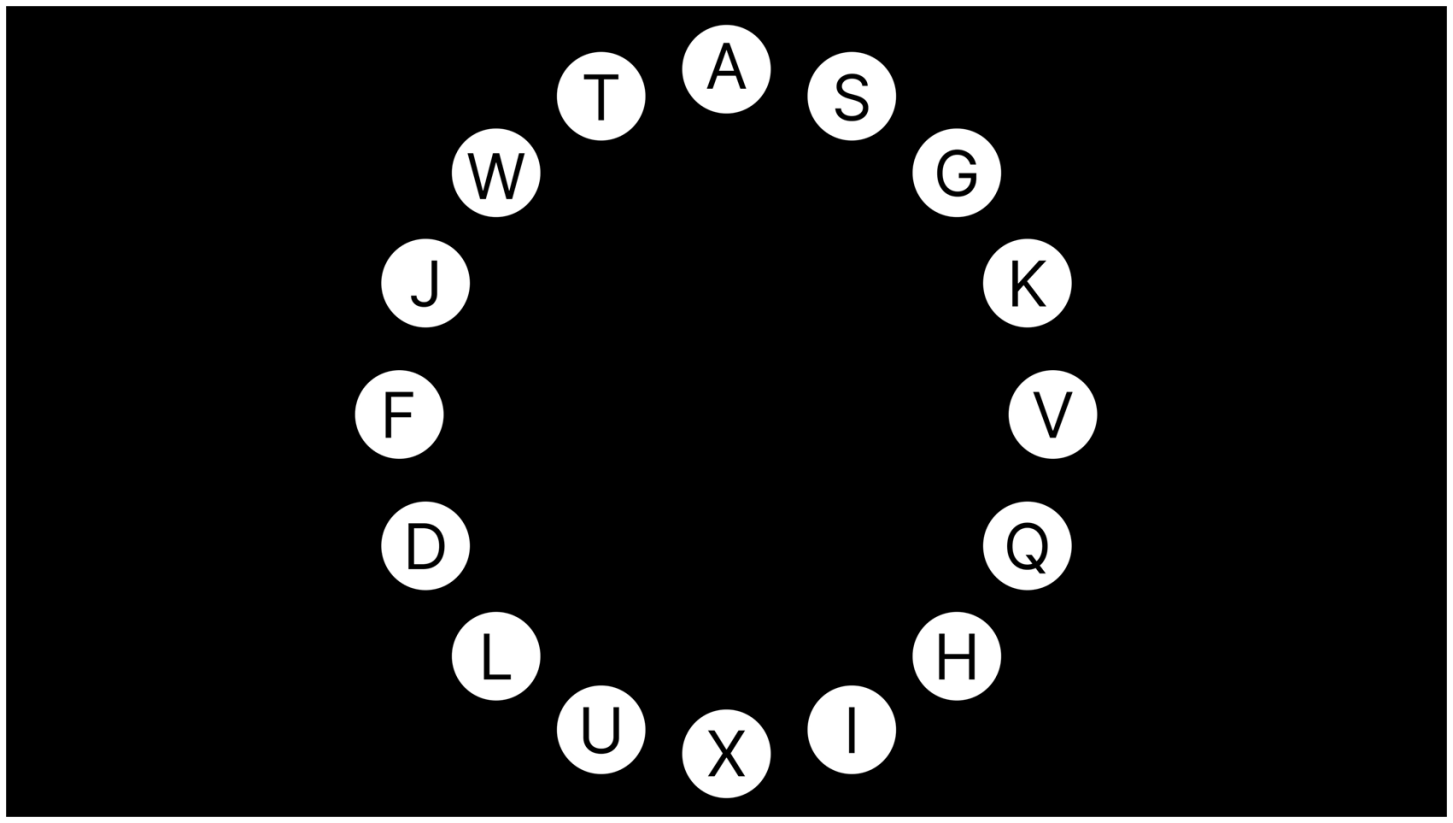
The experimental interface.

The task in this experiment was to select and trigger interactive controls in the interface using a multi-channel eye-control system under the Magilock mechanism. The experimental interface is shown in Figure 3, where 16 white circular icons with capital letters are the interactive controls used in this experiment and are evenly arranged around the center of the screen. In each trial, the letters on the controls appeared randomly and differently, but there must be a control with the letter A. The task of the participants was to search for and trigger the control with the letter A. This experiment was a one-factor, within-subject experiment, and the independent variable was the lock time in the Magilock mechanism. In this experiment, there were five levels of the control lock time (200, 300, 400, 500, and 600 ms), and each level was repeated for 16 trials. The dependent variables in this experiment were the participants’ correct rate of completing the control selection task and completion time. A total of 20 participants participated in this experimental research, all of whom were postgraduate students of Southeast University, aged 24–30 years. The study was approved by the medical ethics committee of the First People’s Hospital of Xuzhou (Affiliated Hospital of China University of mining and Technology), and all of the subjects signed an informed consent form.

### 4.3 Parameter setting basis

Because this study is related to the selection of controls in multi-channel eye-control systems, in the design of the experimental interface, we referred to Fitts’ law research paradigm specified in ISO-9241-9. That is, circular controls uniformly surround the center of the interface, which is also a common control layout pattern used in such research interfaces (Rajanna and Hammond, 2022). In the interface and control color design, the experimental interface in this study used black as the background color and white as the color of the interactive controls, which are easily recognized by the user.

The control size of the experiment was set based on the research findings of Niu et al. (2019) for the optimal control size in an eye-control system. In this study, the distance between the participants and experimental interface was kept at 65–80 cm, and the diameter of the circular control was set to 121px after calculation based on the findings of Niu et al. (2019).

In setting the lock time level, considering the human reaction ability (Luce, 1986), the minimum value of the lock time was set to 200 ms. In existing research on the control triggering time in eye-control systems, the recommended eye gaze dwell times are different because of the differences in the research methods and systems used, mostly in 250–1,000 ms (Elmadjian and Morimoto, 2021). The eye gaze dwell time may decrease when new mechanisms are used, such as when the gaze is combined with an eye blink, and the recommended dwell time is 400 ms (Ma et al., 2023). Therefore, as the independent variable, the lock time in this experiment was set to five levels: 200, 300, 400, 500, and 600 ms.

### 4.4 Procedures of the experiment

The eye tracker was calibrated before the start of the experiment. After calibration, the participants were required to perform a practice experiment to familiarize themselves with the experimental task and operation procedure. After the practice task and short break, the participants began the experiment. The experimental procedure was as follows:

1. In the first step, a ‘+’ was presented in the center of the screen, and the participant needed to gaze at the ‘+’ for 1,000 ms to enter the next interface. This step ensured that the participant’s gaze point was located at the center of the screen at the beginning of each trial.
2. In the second step, 16 circular controls surrounding the center of the screen appeared, and the participant was required to search for the control with the letter A on it.
3. In the third step, the participant needed to keep looking at the control with the letter A. The control started locking and entered the locking state.
4. In the fourth step, the participant was required to keep looking at the control to reach the lock time, and the control entered the locked state. Then, the participant was required to trigger the control through the hand channel by pressing the ‘space’ key on the keyboard.
5. In the fifth step, the control was triggered and the experiment entered the ‘blank’ interface for 1,000 ms to eliminate the visual residue of the participant. If no control was triggered within 10 s, the experiment would also enter the ‘blank’ interface. After the ‘blank’ interface lasted for 1,000 ms, the trial ended and the participant was moved to the next trial.

**Figure 4:**
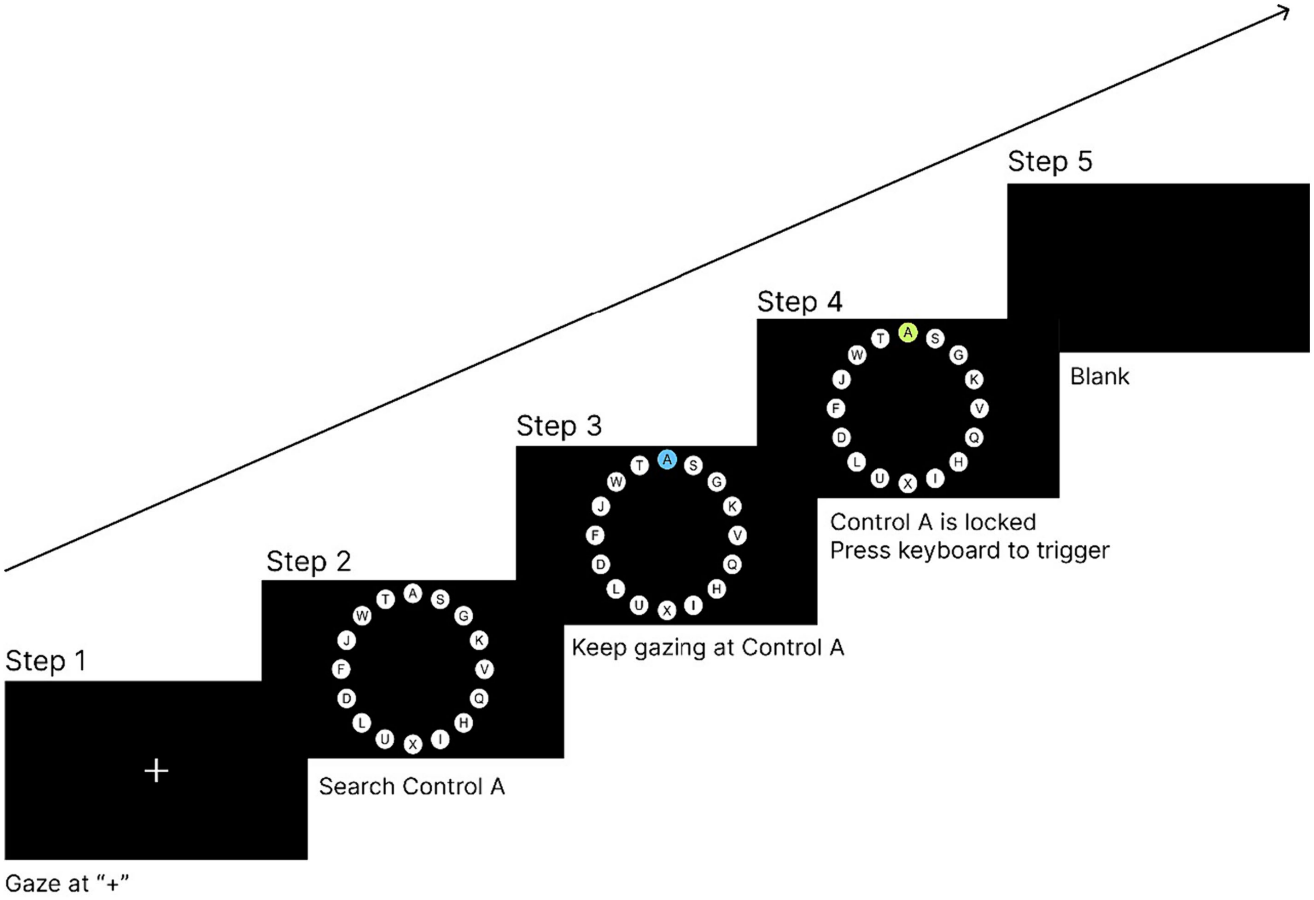
Procedure of one experimental trial.

**Figure 5:**
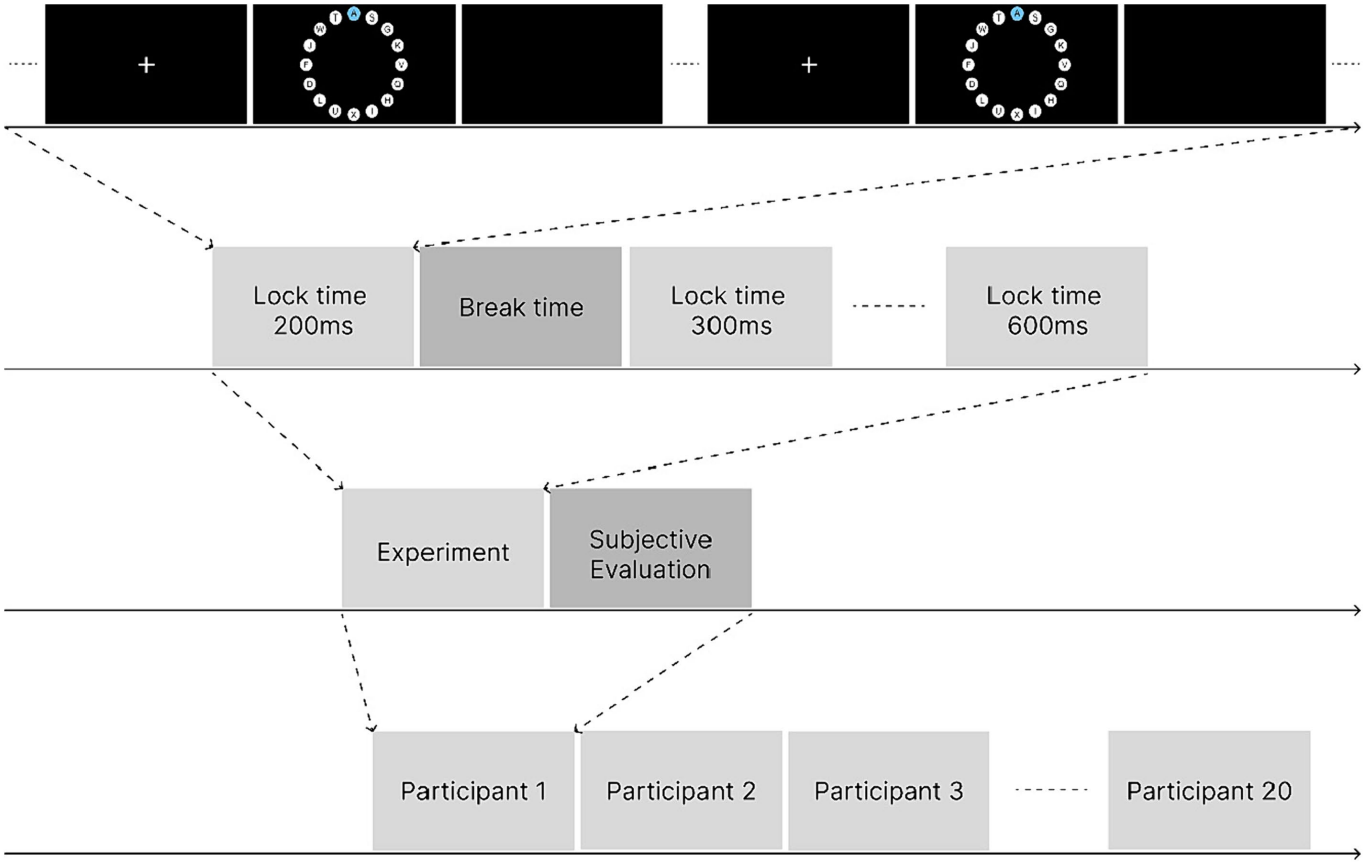
Experimental flow.

Figure 4 illustrates the process of a single experimental trial. The experimental program recorded the participants’ completion time and correctness of the task in each trial in the background for subsequent data analysis. This experiment was a one-factor, within-subject experiment, and each participant took part in all the five lock time experimental levels from 200 to 600 ms. There were 16 trials at each lock time level; therefore, each participant performed a total of 16*5 trials. At the end of the experiment, the participants were asked to complete a SUS usability scale to subjectively evaluate the locking duration at different levels. Before evaluating different lock time levels, the participants undertook the control selection task two more times at the evaluated lock time level to refamiliarize themselves with the interaction experience at that level. The overall flow of the experiment is shown in Figure 5.

### 4.5 Results

In this experiment, the time from the appearance of the control to its triggering in each trial was referred to as the task completion time, and the number of trials that correctly completed the task in proportion to the total number of trials was referred to as the success rate of the experiment.

According to the data recorded by the experimental program, in 20 (number of participants)*5 (number of lock time levels)*16 (number of trials at each level), a total of 1,600 instances of control triggering, there were only 22 cases of failed or incorrect triggering, and the overall control triggering success rate in this experiment was 98.6%.

**Figure 6:**
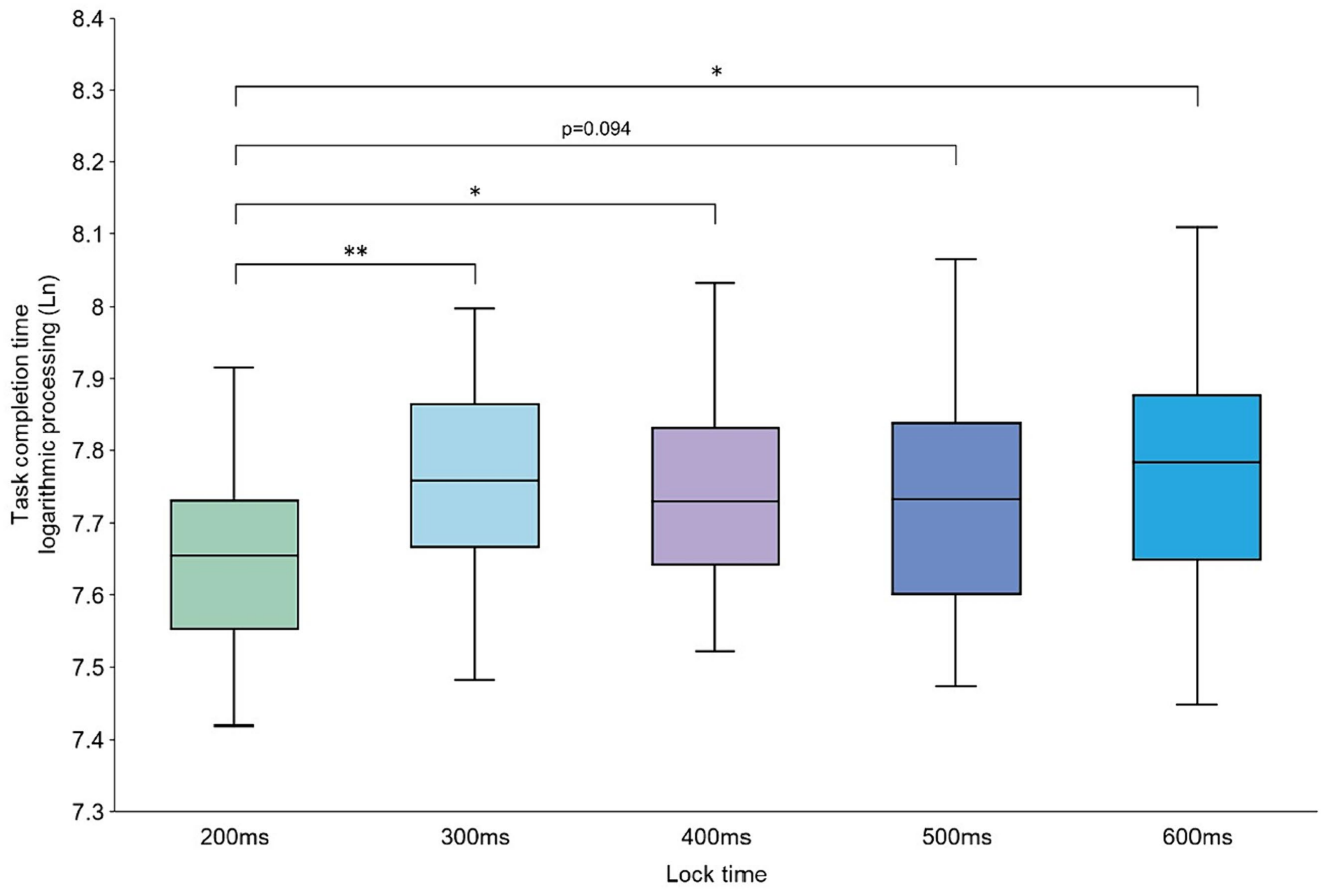
Average task completion time at the 200–600 ms lock time levels.

For the task completion time, the data on failed tasks were filtered out and the outliers were removed by a 3σ method. Then, the average task completion times of the 20 participants at different experimental levels were obtained and analyzed. Since the data did not conform to a normal distribution, we first log-transformed the data and then analyzed the transformed data using ANOVA. We found that the mean task completion times of the participants’ control selection tasks at different lock time levels were significantly different [*F*(4,76) = 2.630, *p* = 0.041]. Furthermore, a two-by-two paired t-test for the data was performed at different lock time levels, and we found that the mean task completion time of the participants at the 200 ms level was significantly different from those at the 300, 400, and 600 ms levels (*p* < 0.05) and borderline significant compared with the mean task completion time at 500 ms (*p* = 0.094). The results of the analysis are shown in Figure 6.

**Figure 7:**
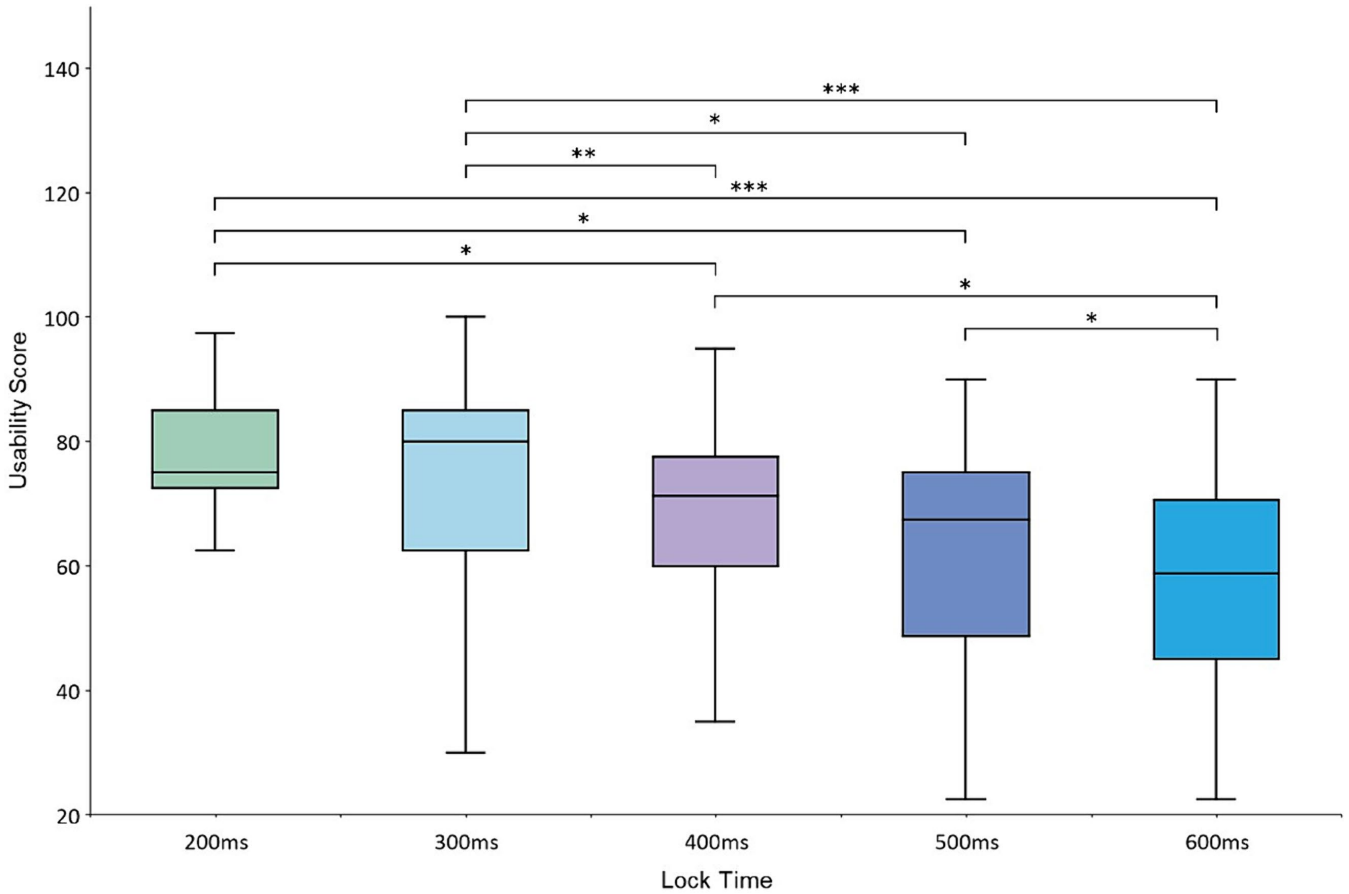
Average usability scores at the 200–600 ms lock time levels.

For the subjective evaluation of the participants, the mean SUS scores at the 200–600 ms lock time levels were 73.1, 74.5, 67, 63.5, and 57.6, respectively. ANOVA of the mean SUS scores showed that the scores at different lock time levels were significantly different [*F*(4,76) = 6.535, *p* < 0.001]. Furthermore, a two-by-two paired *t*-test for the scores was performed at different levels, which showed that the SUS scores at the 200 and 300 ms lock time levels were significantly different from the scores at the 400 and 500 ms levels (*p* < 0.05) and the 600 ms level (*p* < 0.001). The SUS scores at the 400 and 500 ms lock time levels were significantly different from those at the 600 ms level (*p* < 0.05). The results of the analysis are shown in Figure 7.

### 4.6 Discussion

In this experiment, the average correct rate of the participants in the control selection task under the Magilock mechanism was 98.7%, which is higher than most of the present eye-control interaction modes, such as gaze-triggered and blink-triggered combinations of eye-movement interaction modalities or multi-channel eye-control systems (Elmadjian and Morimoto, 2021; Ma et al., 2023; Wang et al., 2024). This demonstrates that the interaction success rate for key tasks improved after the Magilock mechanism was introduced into the multi-channel eye-control system. The reason for the increased success rate of the Magilock interaction mechanism compared with other interaction modes may be that the use of the locked state avoids disorders of the eye and hand channels. Magilock requires the participants to gaze at the control in the locked state before triggering the control by pressing the keyboard with their hands, which prevents the user’s hands from triggering the control before their eyes locate the control correctly. Even if the user presses the keyboard in advance, controls that are not in the locked state are not triggered. This may also be consistent with the finding for a related single-channel eye-control system that an additional confirmation step effectively avoids the Midas touch problem and that the two-step mechanism of ‘lock + confirmation’ effectively improves the success rate of control triggering (Tien and Atkins, 2008).

Analysis of the data on the completion time for the control selection task showed that when the lock time of Magilock was 200 ms, the participants completed the task in a significantly shorter time than at the other levels. There were no significant differences in the task completion times at the other lock time levels. The time the participants needed to trigger the target control increased with increase in the lock time, and that is why the task completion time of the participants at the 200 ms lock time level was significantly lower than the other lock time levels in this study. There were no significant differences in the task completion times at the four lock time levels of 300–600 ms. This may be due to the large number of controls in the experimental interface, which increased the difficulty of searching for target controls. The time required to search for the target control was considerably longer than the lock time, resulting in a non-significant difference in the participants’ task completion times at the four lock time levels of 300–600 ms. Furthermore, increase in the lock time would lead to increase in the time that the participants waited for the control to be locked, which may have led the participants to press the keyboard before the control was locked. This is because the participants in this study would not fail the task if they pressed the keyboard when no controls were locked. When the participants issued a trigger command while the control was in the process of locking, they only needed to issue the trigger command once more and the control would be successfully triggered. This is because the control changes from the locking state to the locked state during the two trigger commands. When the lock time was shorter, the participants tended to issue the trigger command after recognizing that the control had entered the locked state (i.e., after the control had changed from blue to green). However, when the lock time increased, the participants may issue the control trigger command earlier due to the time gap between the brain issuing the action command and the hand executing the action (Yi-yan et al., 2023), as they expected to utilize this time gap to save the control trigger time. The participants’ action strategies to trigger the control differed at different lock time levels, and this may also be the reason for the non-significant difference between the task completion times at the 300–600 ms lock time levels.

In the subjective evaluation analysis of the participants, the average SUS availability scores for the lock times of 200 and 300 ms were significantly higher than the other lock times and had better usability. There was no significant difference between the scores at the 200 and 300 ms lock time levels. This may be due to the fact that, in the Magilock mechanism, the trigger of the control needs to go through a two-step process of gaze locking and hand triggering, as the control is not immediately triggered after being locked; thus, the possibility of the control being mistakenly triggered is very small. A longer lock time does not affect the correct rate of control triggering but affects the efficiency of the interaction process. A longer lock time means that the participant waits longer for the control to be locked before triggering it, which reduces user interaction experience. The lock time levels of 200 and 300 ms were shorter and therefore received higher usability scores from the participants. As the lock time increased, the usability scores for the system decreased because the 400–600 ms locking process was too long for the participants. This was confirmed by communicating with the participants at the end of the experiment.

Both ergonomic experiments and the participants’ subjective evaluations suggest that it is more appropriate to set a shorter locking duration for Magilock. At the lock time level of 200 ms, the average time taken by the participants to complete the experimental task was significantly shorter than at other levels, and the average usability scores given by the participants were higher at the lock time levels of 200 and 300 ms. This may be due to the fact that the Magilock mechanism has a higher interaction accuracy, and the participants tend to spend less time to complete each interaction task. When the lock time of the Magilock mechanism was set to 200 ms, the participants’ usability scores were higher and the average time to complete the experimental tasks was shorter. Therefore, we recommend setting the lock time of the Magilock mechanism to 200 ms.

### 4.7 Conclusion

When the lock time was set to 200 ms, the time required for the participants to complete the control trigger task was shorter and the system usability scores given by the participants were higher. Therefore, 200 ms can be used as a reference value for setting the lock time in the Magilock mechanism.

## 5 Magilock unlock time research

### 5.1 Purpose

When browsing for information, users may keep their eye gaze point on a control for a while, which locks the control. When users want to trigger a control, they may need to unlock the locked nontarget control first. If the unlock time is too long, it affects interaction efficiency and user experience. If the unlock time is too short, the control may be unlocked unexpectedly due to the drift of the user’s gaze point, which may lead to failure of the control trigger. Therefore, while the former experiment examined the lock time of the Magilock mechanism, this experiment further explored the unlock time of the Magilock mechanism. It is expected that the optimal unlock time for the Magilock mechanism will be determined to ensure the efficiency and experience of user interaction.

### 5.2 Design

This experiment investigated the unlock time in the Magilock mechanism. The task required the participants to first lock the control adjacent to the target control, then unlock the adjacent control, and finally lock the target control again and trigger it. The experimental interface and participants taking part in this experiment were the same as those in Section 4.2. This experiment was also a within-subject, one-factor experiment, and the independent variable was the unlock time of the control. Based on the results presented in Section 4.1, the lock time in this experiment was set to 200 ms. With reference to the variable levels of the lock time in Section 4.3, the unlock time of the control in this experiment was set to five levels: 200, 300, 400, 500, and 600 ms. Each participant repeated the experiment 16 times at each level. The dependent variables of the experiment were the correct rate and completion time of the experimental task. These data were recorded in the background using an experimental program.

### 5.3 Procedures of the experiment

Preparation prior to the formal experiment was the same as that described in Section 4.3. After the practice experiment, the participants could start the formal experiment, and the procedure of each trial in the formal experiment was as follows:

1. In the first step, a ‘+’ was presented in the center of the screen. The participants were asked to gaze at the ‘+’ for 1,000 ms to enter the next interface.
2. In the second step, 16 circular controls surrounding the center of the screen appeared, and the participants were required to find and gaze at the control adjacent to the target control A until it was locked.
3. In the third step, the participants were required to move their eye gaze points away from the control. The control was unlocked after the gaze point was removed for the Magilock unlock time. The participants were required to move their eye gaze point to the target control A and keep gazing at it.
4. In the fourth step, the target control A was locked after a continuous gaze for 200 ms. The participants needed to trigger the target control through the hand channel by pressing the ‘space’ on the keyboard.
5. In the fifth step, after the control was triggered, the experiment entered into the ‘blank’ interface, which lasted for 1,000 ms and was used to eliminate the participant’s visual field residue. If no control was triggered within 10 s, the experiment would enter into the ‘blank’ interface. After the ‘blank’ interface lasted for 1,000 ms, the current trial ended and the participant moved to the next trial.

**Figure 8:**
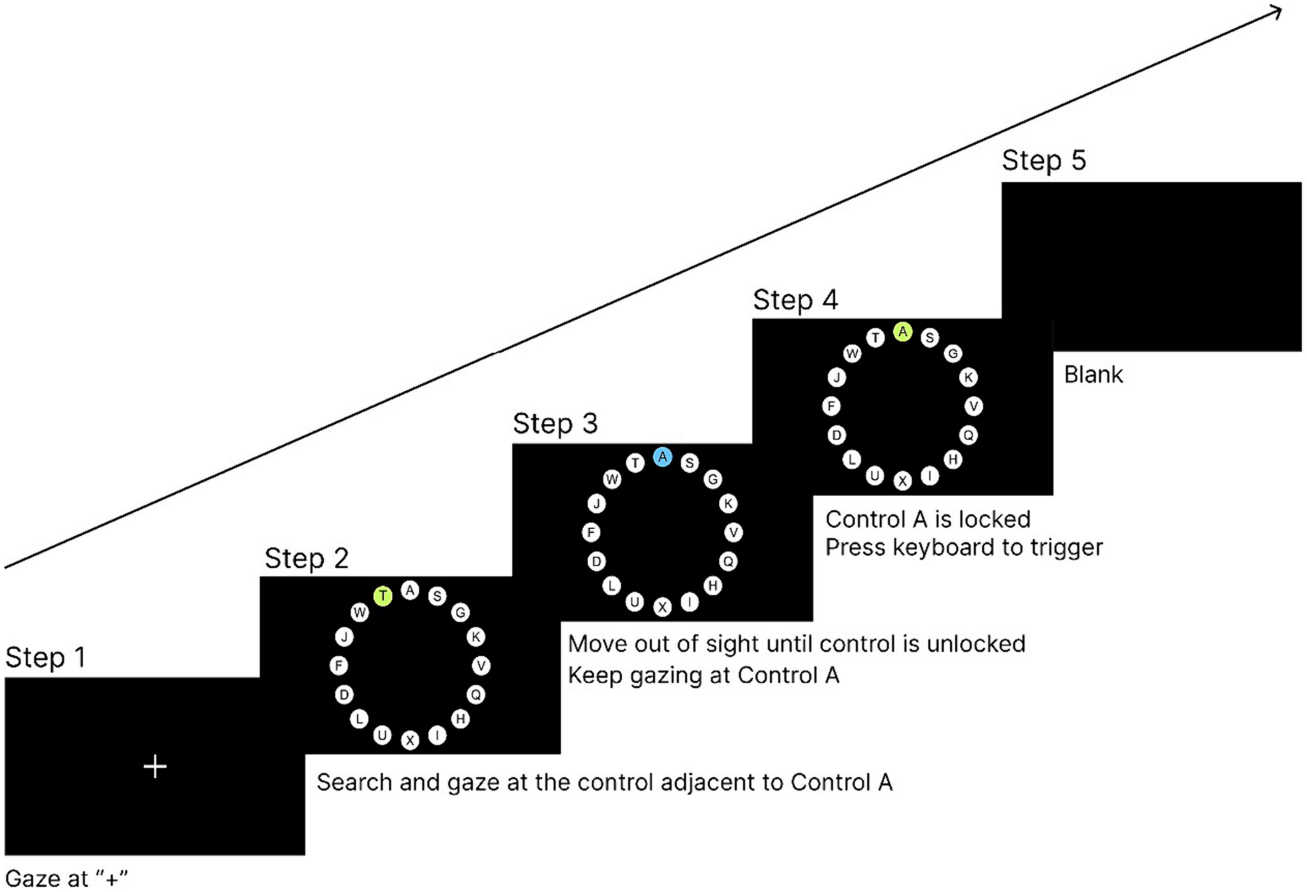
Procedure of one experimental trial.

Figure 8 shows the process of a single experimental trial. Each participant was required to complete an SUS scale for the unlock time levels at the end of the experiment.

### 5.4 Results

In the total of 1,600 control trigger tasks of 20 (number of participants)*5 (number of unlocking time levels)*16 (number of trials in each level), the total number of task failures was 60. The overall success rate of the control trigger was 96.3%.

**Figure 9:**
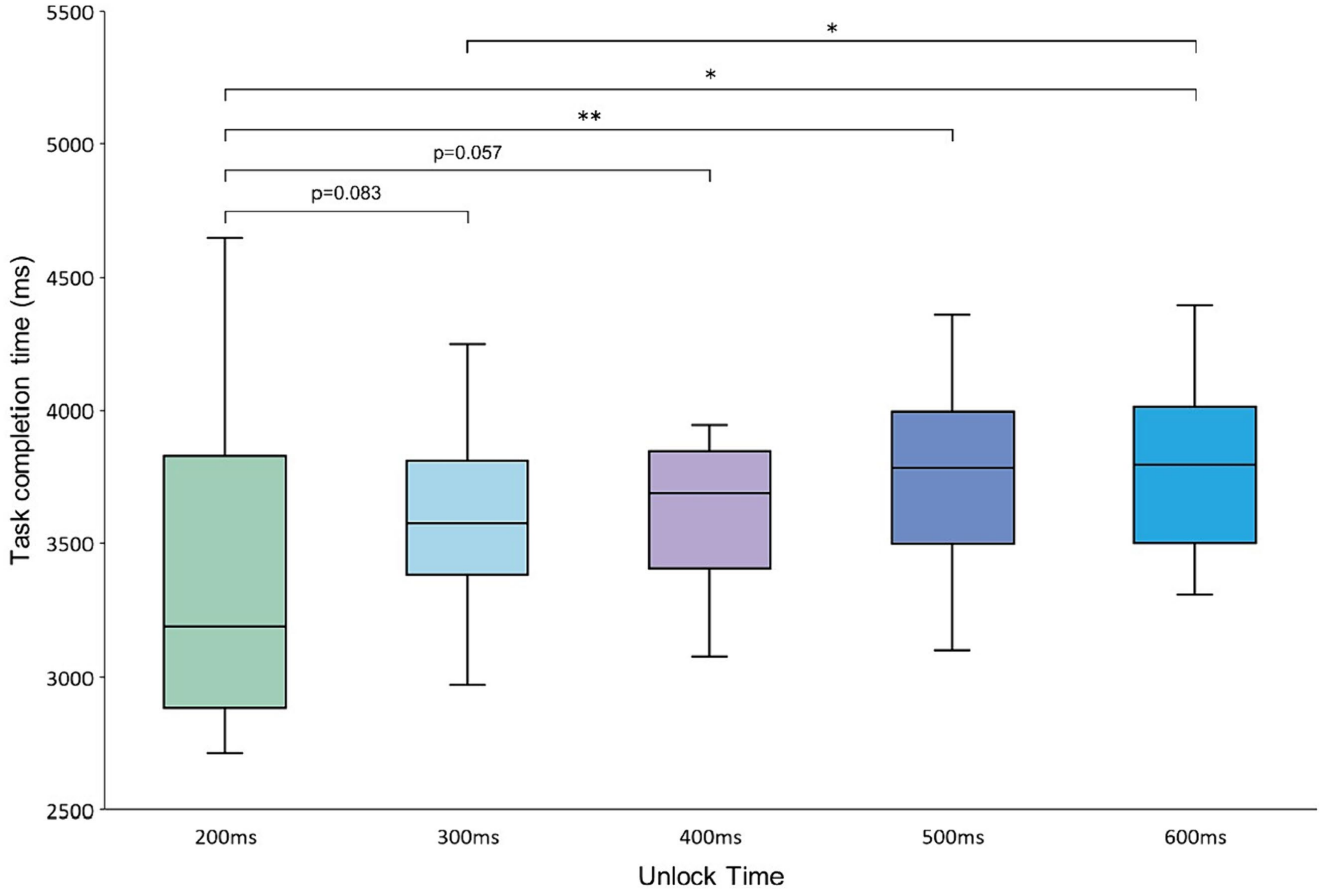
Average task completion time at the 200–600 ms unlock time levels.

For the task completion time, the data on failed tasks were filtered out and the outliers were removed by a 3σ method. Then, the average task completion times of the 20 participants at different experimental levels were obtained and analyzed. ANOVA revealed that the completion time of the task was significantly different at different unlock time levels [*F*(4,76) = 4.537, *p* = 0.003]. Furthermore, a two-by-two paired *t*-test for the data was performed at different unlock time levels. The average task completion time of the participants at the 200 ms level was significantly different from that at the 500 and 600 ms levels (*p* < 0.05) and borderline significant compared with the data at the 300 ms and 400 ms levels (*p* = 0.083, *p* = 0.057). The difference between the average task completion times at the 300 and 600 ms unlock time levels was significant (*p* < 0.05). The results of the analysis are shown in Figure 9.

**Figure 10:**
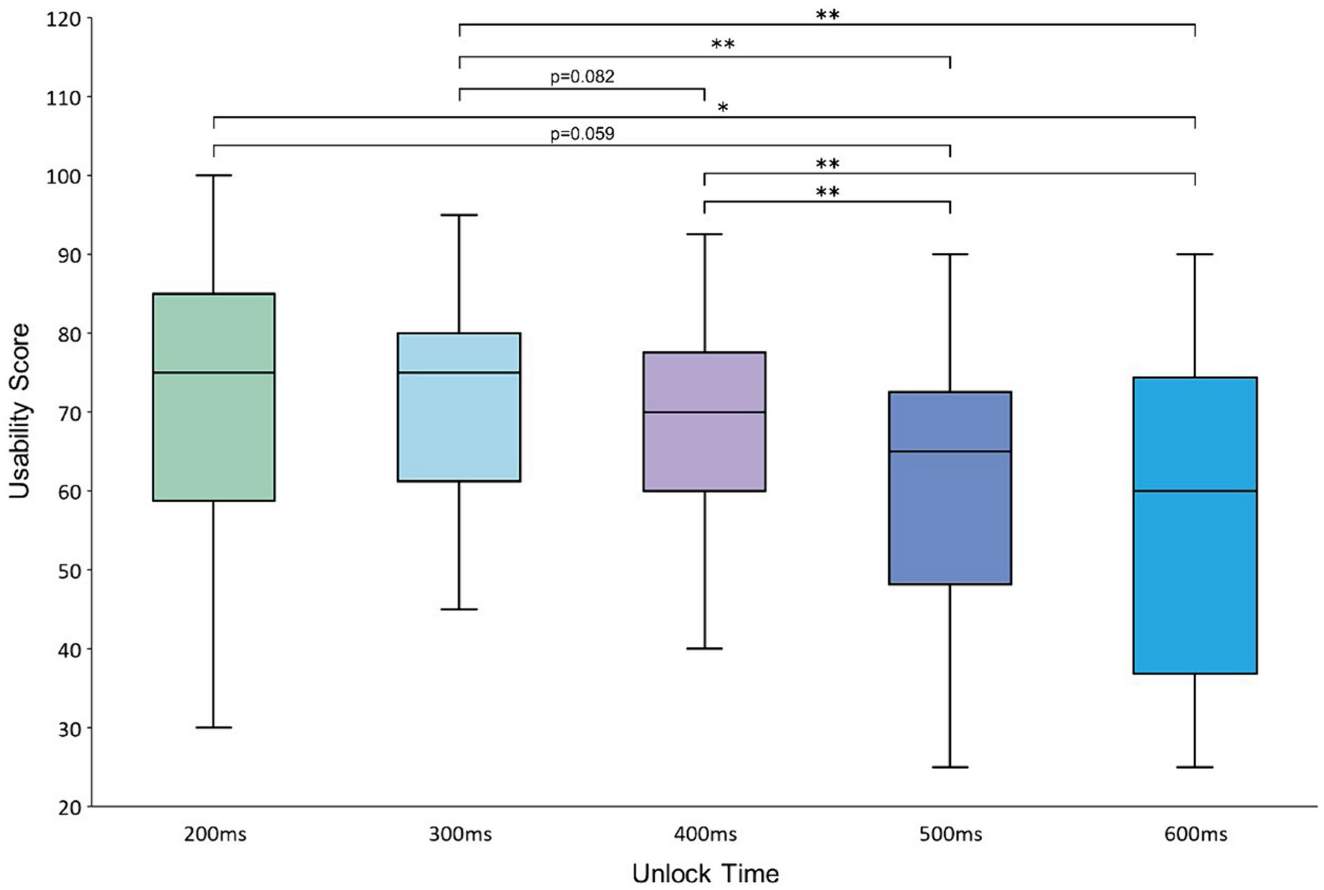
Average usability scores at the 200–600 ms unlock time levels.

For the subjective evaluation, the SUS scores at the 200–600 ms unlock time levels were 71.3, 71.8, 68, 60, and 56.5, respectively. ANOVA of the SUS scores given by the participants showed that there was a significant difference between the scores at different unlock time levels [*F*(4,76) = 6.409, *p* < 0.001]. Furthermore, a two-by-two paired *t*-test for the data of the scores was performed at different levels, and there was a significant difference between the SUS scores at the 200 and 600 ms levels (*p* < 0.05). The scores at the 200 ms level were borderline significant compared with those at the 500 ms level (*p* = 0.059). The scores at the 300 ms unlock time level were significantly different from those at the 500 and 600 ms levels (*p* < 0.01) and borderline significant compared with those at the 400 ms level (*p* = 0.082). The scores at the 400 ms unlock time level were significantly different from those at the 500 and 600 ms levels (*p* < 0.01). The results of the analysis are shown in Figure 10.

### 5.5 Discussion

The average correct rate of the participants’ control selection task in this experiment was 96.3%, which is a decrease compared to the 98.7% correct rate in the experiment in Section 4.1. This may be due to the fact that the interaction task in this experiment was more complex, as the participants had to perform an additional locking and unlocking operation before triggering the target control (Chin and Barreto, 2007; Glaholt and Reingold, 2009). The 96.3% correct rate in this experiment also proves that the Magilock mechanism is highly reliable and usable, as it ensures that the user can quickly trigger the target control even if the nontarget is incorrectly locked in a complex interaction flow.

For the task completion time, the task in this experiment was more complex, which required the user to lock and then unlock the controls adjacent to the target control before locking and triggering the target control. When the experimental task was completed, the complex interaction process required more interaction time and effort from the participants, and it was necessary to shorten the length of the time required for the interaction task (Argelaguet and Andujar, 2013; Zhou et al., 2020). Analysis of the data of task completion at each level of the unlock time showed that as the unlock time increased in the Magilock mechanism, the average time taken by the participants to complete the experimental task also increased. This means that setting a shorter unlock time for Magilock can effectively shorten the interaction time of the participants. In the significance analysis, the task completion time at the unlock time level of 200 ms was significantly lower than that at the unlock time levels of 500 and 600 ms and also marginally significant compared with the 300 and 400 ms levels. Thus, setting the unlock time to 200 ms may effectively improve the interaction efficiency of the participants.

For the subjective evaluation of the system’s usability, users tended to give higher SUS scores when the unlock time in the Magilock mechanism was shorter, which was confirmed by the significance analysis of the SUS scores for the system at all levels of the unlock time. The SUS scores of the unlock time levels of 200 and 300 ms were 71.3 and 71.8, respectively, which were higher than the scores at the 400–600 ms levels. This indicates that systems with unlock times set to 200 and 300 ms have higher usability than those with unlock times set to 400, 500, and 600 ms. This may be due to the fact that the Magilock mechanism has a high control selection and trigger success rate, and a longer unlock time would have little effect on increasing the control trigger success rate. Moreover, the operation of unlocking a control often occurs when the locked control is not the user’s target control. Therefore, the user may expect to change the state of the control quickly. A long unlock time reduces interaction efficiency. If the unlock time of a control is too long, it may lead to a situation in which the user may have already shifted the gaze point to the target control, but the target control cannot be locked because the locked nontarget control has not yet been unlocked, which greatly affects the user’s interaction experience. This was also confirmed through communication with the participants after the experiment.

Combining the results of the experiment and subjective evaluations of the participants, it might be more appropriate to set a shorter unlock time for the Magilock mechanism. When the unlock time was shorter, the average time the participants took to complete the experimental task was shorter, as the average time to complete the task at the 200 ms level was significant or borderline significant compared with other levels. The participants also tended to give higher usability scores when the unlock time was shorter. Both the average correct rate of the experiment and communication with the participants showed that the Magilock mechanism is more reliable and that an excessively long unlock time makes the interaction process insufficiently smooth for the participants, which reduces their experience of using it. When the unlock time of the Magilock mechanism was set to 200 ms, the usability scores of the participants were high and the average time to complete the experimental task was shorter. Therefore, it is recommended to set the unlock time of the Magilock mechanism to 200 ms.

### 5.6 Conclusion

When the lock time was set to 200 ms, the time required for the participants to complete the control trigger task was shorter and the system usability scores given by the participants were higher. Therefore, 200 ms can be used as a reference value for setting the lock time in the Magilock mechanism.

## 6 Conclusion

In this study, we proposed a control trigger mechanism called Magilock for a multi-channel eye-control system. Magilock adds a gaze-locking step in the process of two-channel cooperation between the eye channel positioning the control and the hand channel triggering the control. Under the Magilock mechanism, controls in the eye-control system can only be triggered after they are locked by gaze, which effectively improves the success rate of the target control triggering in the system. Magilock is designed to be applied to low-frequency key commands with high error-correction costs, for which the user often requires a higher trigger success rate. The proposed Magilock mechanism ensures the user’s interaction experience when executing key commands in the eye-control system. We also conducted ergonomic experiments on the lock and unlock times in the Magilock mechanism. Both ergonomic experiments and subjective evaluations of the participants showed the recommended lock and unlock time of Magilock, which further ensures the interaction efficiency and experience of users when using the multi-channel eye-control system under this mechanism. The design concept of Magilock, which adds a locking mechanism between the eye-control channel and other interactive channels, can be generalized to different systems and provides a new idea for the design of key command triggering methods in multi-channel eye-control systems. The main conclusions of this study are as follows.

1. A one-factor, within-subject ergonomic experiment was conducted to investigate the lock time of the Magilock mechanism. The independent variable of the experiment was the lock time at five levels: 200, 300, 400, 500, and 600 ms. The task completion time was shorter and the SUS scores were higher when the lock time was 200 ms; thus, 200 ms was recommended as the lock time for the Magilock mechanism.
2. A one-way, within-subject ergonomic experiment was conducted to investigate the unlock time of the Magilock mechanism. The independent variable was the unlock time, and there were five levels of the unlock time: 200, 300, 400, 500, and 600 ms. The participants’ task completion time was shorter and the usability scores were higher when the unlock time was 200 ms. Therefore, 200 ms was recommended as the unlock time for the Magilock mechanism.

This paper studies the visual and tactile interaction mode of “eye control locking & controller triggering” in eye-control systems. A reasonable collaborative mechanism of visual/tactile interaction is designed in this work, which improves the efficiency of eye control interaction. The research results can provide important reference for establishing a scientific visual and tactile interaction design method for eye-control systems that integrates entity controllers.

## 7 Limitations and future works

The findings of this study can be used in multi-channel eye-control systems. However, due to certain objective factors and conditions, there are still some limitations to this study that need to be considered and further explored.

1. The participants in this study were all 24–30-year-old postgraduate students in school. There may be significant differences in the eye movement interaction performance between different age groups. Thus, the effect of age on the Magilock mechanism requires further investigation.
2. Different control shapes, control sizes, and interface layouts may affect user interaction performance. The color stimulus of the interface and controls and feedback form of the control may also affect the results of the experiment, which needs to be further investigated.
3. The command output form used in this study was to press the keyboard. When other interaction channels are used as command output channels with the eye-control system, the results of this study may not be suitable and require further investigation.
4. This study used an eye tracker to collect user gaze points and developed experimental designs using the Tobii Unity SDK for Desktop. The eye tracker used in the experiment is Tobii 5 (sampling rate: non-interlaced gaze at 33 Hz). In the future, we can try to conduct research work with a higher sampling rate eye tracker device (such as Tobii Pro Fusion, sampling rate 250 Hz) to further improve the accuracy of experimental results and reduce system latency.

Future research should consider introducing the Magilock interaction mechanism into different multi-channel eye-control systems. Moreover, future research should investigate the adaptability of the Magilock mechanism with other commands of the eye-control system, try to apply the Magilock mechanism to the drag-and-drop, box-select, or some shortcut commands in the eye-control system, and develop the corresponding interaction prototypes.

## Data availability statement

The raw data supporting the conclusions of this article will be made available by the authors, without undue reservation.

## Ethics statement

The studies involving humans were approved by Medical ethics committee of the First People’s Hospital of Xuzhou (Affiliated Hospital of China University of mining and Technology). The studies were conducted in accordance with the local legislation and institutional requirements. The participants provided their written informed consent to participate in this study.

## Author contributions

NY-F: Funding acquisition, Methodology, Project administration, Supervision, Writing – review & editing. HJ-X: Investigation, Methodology, Software, Writing – original draft. LJ: Conceptualization, Formal analysis, Validation, Writing – original draft.
